# In Vitro and In Vivo Antibacterial Activity of Gliotoxin Alone and in Combination with Antibiotics against *Staphylococcus aureus*

**DOI:** 10.3390/toxins13020085

**Published:** 2021-01-23

**Authors:** Patricia Esteban, Sergio Redrado, Laura Comas, M. Pilar Domingo, M. Isabel Millán-Lou, Cristina Seral, Sonia Algarate, Concepción Lopez, Antonio Rezusta, Julian Pardo, Maykel Arias, Eva M. Galvez

**Affiliations:** 1 Fundacion Instituto de Investigacion Sanitaria Aragon (IIS Aragon), Biomedical Research Centre of Aragon (CIBA), 50009 Zaragoza, Spain; patri.estb@gmail.com (P.E.); pardojim@unizar.es (J.P.); 2 Instituto de Carboquımica ICB-CSIC, 50018 Zaragoza, Spain; sergio-redrado@hotmail.com (S.R.); lcomasc@hotmail.com (L.C.); mpdomingo@icb.csic.es (M.P.D.); 3 Department of Microbiology, Hospital Universitario Miguel Servet, IIS Aragón, 50009 Zaragoza, Spain; isiml68@gmail.com (M.I.M.-L.); concepta963@icloud.com (C.L.); arezusta@unizar.es (A.R.); 4 Department of Microbiology, University Clinic Hospital Lozano Blesa, 50009 Zaragoza, Spain; cseral@salud.aragon.es (C.S.); sonialgarate@gmail.com (S.A.); 5 Department of Microbiology, Pediatrics, Radiology and Public Health, University of Zaragoza, 50009 Zaragoza, Spain; 6 Aragon I+D Foundation (ARAID), 50018 Zaragoza, Spain

**Keywords:** gliotoxin, *Staphylococcus aureus*, MRSA, VISA, vancomycin, synergism, resistance, antibiotic

## Abstract

Multidrug-resistant bacteria such as methicillin-resistant *Staphylococcus aureus* (MRSA) is one of the major causes of hospital-acquired and community infections and pose a challenge to the human health care system. Therefore, it is important to find new drugs that show activity against these bacteria, both in monotherapy and in combination with other antimicrobial drugs. Gliotoxin (GT) is a mycotoxin produced by *Aspergillus fumigatus* and other fungi of the *Aspergillus* genus. Some evidence suggests that GT shows antimicrobial activity against *S. aureus* in vitro, albeit its efficacy against multidrug-resistant strains such as MRSA or vancomycin-intermediate *S. aureus* (VISA) strainsis not known. This work aimed to evaluate the antibiotic efficacy of GT as monotherapy or in combination with other therapeutics against MRSA in vitro and in vivo using a *Caenorhabditis elegans* infection model.

## 1. Introduction

*Staphylococcus aureus* is a Gram-positive bacterium often present as part of the normal microbiota of the human body [1,2,3]. It is an opportunistic pathogen that after surpassing the skin barrier can cause a variety of systemic and pyogenic infections, acute and chronic infections, and toxin-mediated syndromes [4]. *S. aureus* infections range from skin and soft tissue infections to severe necrotizing pneumonia, life-threatening endocarditis, or bacteremia in adults and children [5,6,7,8]. During the modern antibiotic era, *S. aureus* has evolved the ability to acquire resistance to most antibiotics. Methicillin-resistant *Staphylococcus aureus* (MRSA) emerged in the 1960s [9], being a prevalent and important bacterium that has spread globally and has become a leading cause of both nosocomial and community-acquired bacterial infections [10]. MRSA infections are associated with higher mortality rates than infections caused by methicillin-susceptible *S. aureus* (MSSA) strains [11]. Vancomycin is a glycopeptide antibiotic that inhibits cell wall biosynthesis and remains a drug of choice for the treatment of severe MRSA infections [12,13]. However, in the last 20 years, clinical isolates of vancomycin intermediate-resistant *S. aureus* (VISA, minimum inhibitory concentration (MIC) = 4–8 μg/mL) with reduced susceptibility to vancomycin have emerged, which is associated with persistent infections, treatment failure, and poor clinical outcomes. Additionally, although less frequently, *S. aureus* strains with complete resistance to vancomycin (VRSA, MIC ≥ 16 μg/mL) have been described [14,15,16].

These multidrug-resistant organisms pose a challenge to the current human health care system. Therefore, the development of new treatment strategies to combat resistant *S. aureus* infections is necessary. Renewed efforts are needed for research and development of new antibiotics, or the combination of these with existing treatments for which bacterial resistances are emerging. One of the lines of antibiotics discovery is the exploration of the antibacterial activity of natural products. Microbial secondary metabolites have been studied for their potential benefits to humans and have provided numerous pharmaceutical products. Fungi have the ability to produce a wide variety of secondary metabolites, generally dependent on the stage of fungal development and environmental factors [17]. *Aspergillus fumigatus* and other fungi belonging to the *Aspergillus* genus are especially capable of producing a great diversity of compounds. In fact, they secrete more than 226 secondary metabolites, including epipolythiodioxopiperazines (ETPs), whose best studied member is gliotoxin (GT) [18].

**Figure 1:**
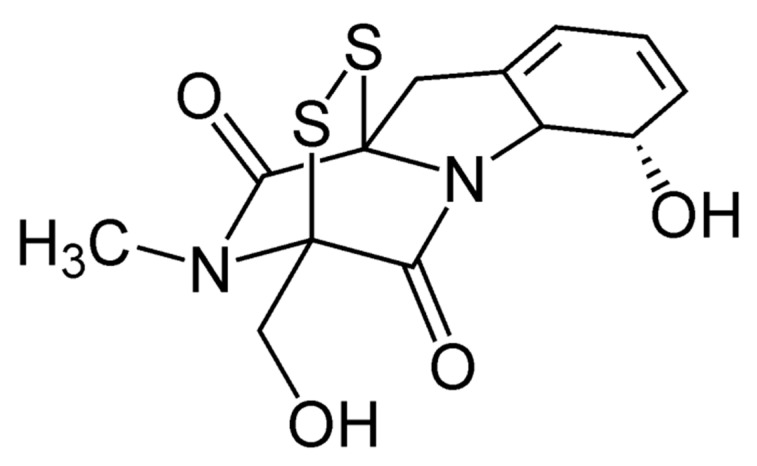
Gliotoxin (GT) structure is characterized by a piperazine ring containing a disulfide bridge. Image obtained from Wikimedia Commons.

**Figure 2:**
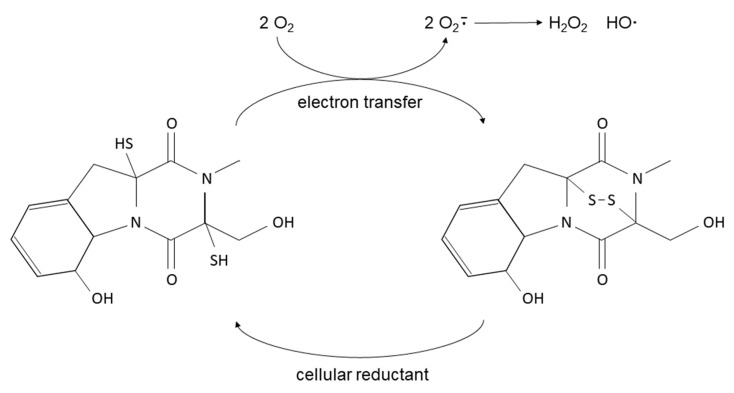
Redox cycle between the reduced (dithiol, left) and oxidized (disulfide, right) forms of GT. The oxidation of GT and presumably other epipolythiodioxopiperazines (ETPs) generates reactive oxygen species since it is capable of reducing molecular oxygen [18].

GT (Figure 1) is characterized by the presence of an internal disulfide bridge in a piperazine ring, which seems to be necessary for most of the biological properties of this compound [19]. This mycotoxin exerts its toxic action by generating reactive oxygen species due to the sulfur contained in the molecule, being able to alternate between a completely reduced dithiol form and a disulfide form (Figure 2) [20].

Furthermore, reduced GT can react with other accessible thiol groups on proteins, inactivating, inhibiting, or denaturing them [18]. Some in vitro studies show that the reactive oxygen species produced by GT are capable of causing DNA damage [21,22,23].

There is currently limited scientific evidence on the use of GT as an antibiotic. The antibacterial action of GT has been studied against *Escherichia coli* and *S. aureus*. It is suggested that GT could cause damage to *E. coli* DNA [24] and that it exhibits antibiotic effect for both microorganisms, being more effective against *S. aureus* [25,26].

Here we have extended these studies and used a MRSA/VISA strain to study the antibacterial activity of GT alone and in combination with vancomycin. We found that GT has a potent in vitro antibiotic effect against this strain and that its combination with vancomycin shows a very good synergy at lower concentrations than single compounds. We also carried out an in vivo study using *Caenorhabditis elegans* as a model for MRSA infection, where the combined treatment demonstrated a significant higher survival percentage than the treatment with vancomycin as monotherapy. Therefore, our findings reveal a novel approach to take advantage of the antibiotic activity of GT and its potentiating effect when combined with vancomycin. Thus, a greater antibiotic effect is obtained with a lower concentration of vancomycin, and therefore the possible side effects and the appearance of resistance due to the use of vancomycin could be reduced.

## 2. Results

### 2.1. Antimicrobial Susceptibility of S. aureus against GT

#### 2.1.1. Effect of GT on *S. aureus* Growth

**Figure 3:**
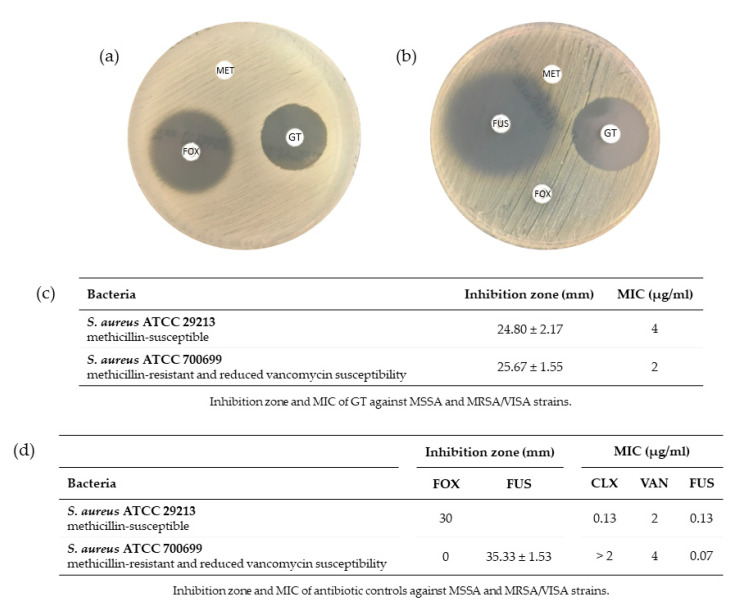
Antimicrobial activity of GT against methicillin-susceptible *Staphylococcus aureus* (MSSA) and methicillin-resistant *S. aureus*/vancomycin intermediate-resistant *S. aureus* (MRSA/VISA) strains. (**a**) *S. aureus* ATCC 29213 strain antibiogram. (**b**) *S. aureus* ATCC 700699 strain antibiogram. A disk containing the methanol solution (MET) with sterile distilled water is located on top of the plates. The disk containing the control antibiotic is placed on the left side of the plate: (**a**) cefoxitin (FOX) 30 μg and (**b**) fusidic acid (FUS) 10 μg. On the right side, the disk impregnated with GT (10 μg) is placed. In the case of resistant *S. aureus* (**b**), there is a fourth disk at the bottom of the plate, where FOX 30 μg disk is located. *n* ≥ 3. (**c**) Table containing the mean ± s.d. of the growth inhibition halos (diameter in mm), as well as the minimum inhibitory concentration (MIC) (µg/mL) calculated as described in Materials and Methods of GT for each strain. (**d**) Table containing the mean ± s.d. of the growth inhibition halos (diameter in mm), as well as the MIC (µg/mL) calculated as described in Materials and Methods of antibiotic controls for each strain: cloxacillin (CLX), vancomycin (VAN) and FUS. *n* = 3.

As a result of antibiograms, the mean of the inhibition halos obtained for each impregnated disk was calculated (Figure 3). All antimicrobials used as controls produced an inhibition halo within the range established by the European Committee on Antimicrobial Susceptibility Testing (EUCAST 2020) specific for each strain. The inhibition halo of the water and methanol disks was 0 mm in all cases. Thus, these results show that the vehicle used to solve GT, methanol, does not exert any antimicrobial action on any of the microorganisms under the tested conditions.

Inhibition halos were clearly observed around GT disks in antibiograms, indicating that GT is capable of negatively affecting the growth of these strains. It is important to point out the growth inhibition effect of GT on MRSA, where the inhibition halo obtained was slightly higher than the halo of the MSSA strain.

#### 2.1.2. Minimum Inhibitory Concentration of GT and Antibiotics against *S. aureus*

In Figure 3c, the values obtained for the MIC of GT in the two strains of *S. aureus* are indicated. It is important to note that the MIC of the MRSA strain (2 µg/mL) was lower than the MIC of the MSSA strain (4 µg/mL), which is consistent with the results of the antibiograms, where the inhibition halo of the strain MRSA was greater than that of MSSA for the same conditions of the experiment.

As shown in Figure 3d, all antimicrobials used as a control for the *S. aureus* ATCC 29132 strain resulted in a MIC consistent with the range established by EUCAST: Routine and extended internal quality control for MIC determination and disk diffusion as recommended by EUCAST, version 10.0, 2020, http://www.eucast.org. Concerning the *S. aureus* ATCC 700699 strain, the susceptibility obtained with the different antibiotics used as controls (Figure 3d) corroborated that it was a MRSA (shows resistance to cloxacillin) and VISA (MIC = 4 µg/mL for vancomycin) strain.

### 2.2. Antimicrobial Synergy of GT in Combination with Anti-Staphylococcal Drugs

**Figure 4:**
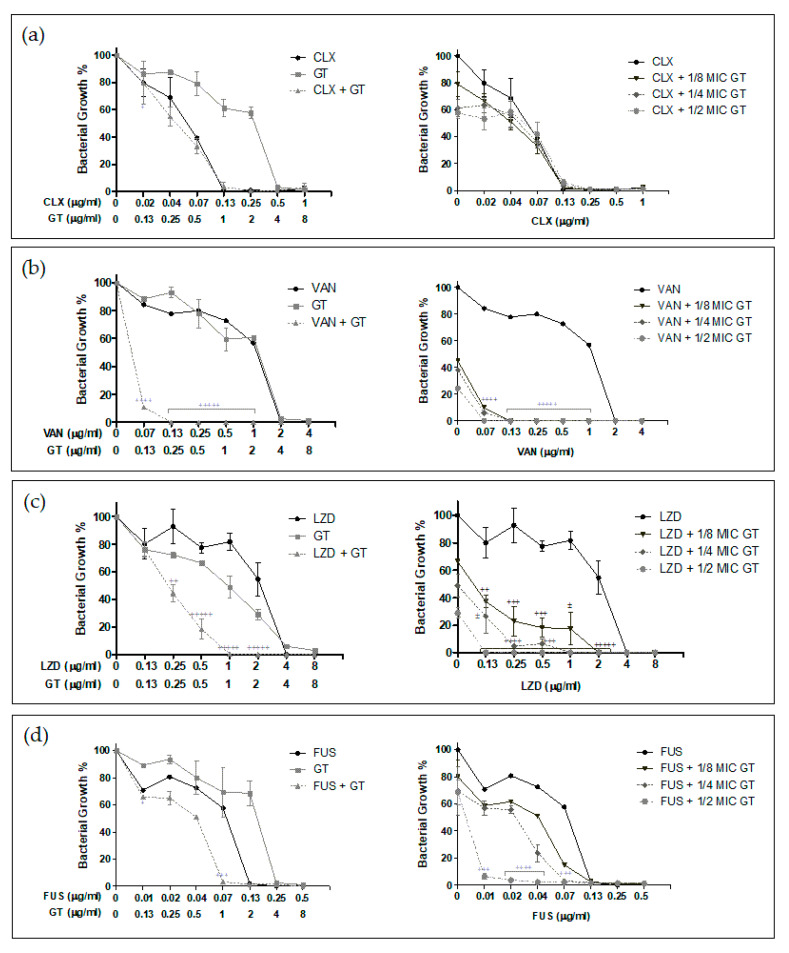
In vitro synergy assay results for MSSA ATCC 29213 strain. The effect of GT and different antibiotics as monotherapy or in combination was tested against *S. aureus* as described in Materials and Methods. (**a**) Combination of GT (GT) and cloxacillin (CLX). (**b**) Combination of GT and vancomycin (VAN). (**c**) Combination of GT + linezolid (LZD). (**d**) Combination of GT + fusidic acid (FUS). For each panel, the concentration of each compound and the mean ± s.d. of the percentage of bacterial growth obtained in the experiments are indicated. The symbols correspond to the different levels of synergy as indicated in Materials and Methods, calculated using the CalcuSyn software, as indicated in Materials and Methods: (±) Nearly additive, (+) Slight synergism, (++) Moderate synergism, (+++) Synergism, (++++) Strong synergism, and (+++++) Very strong synergism. MIC of GT = 4 µg/mLfor *S. aureus* ATCC 29213. *n* = 3.

**Figure 5:**
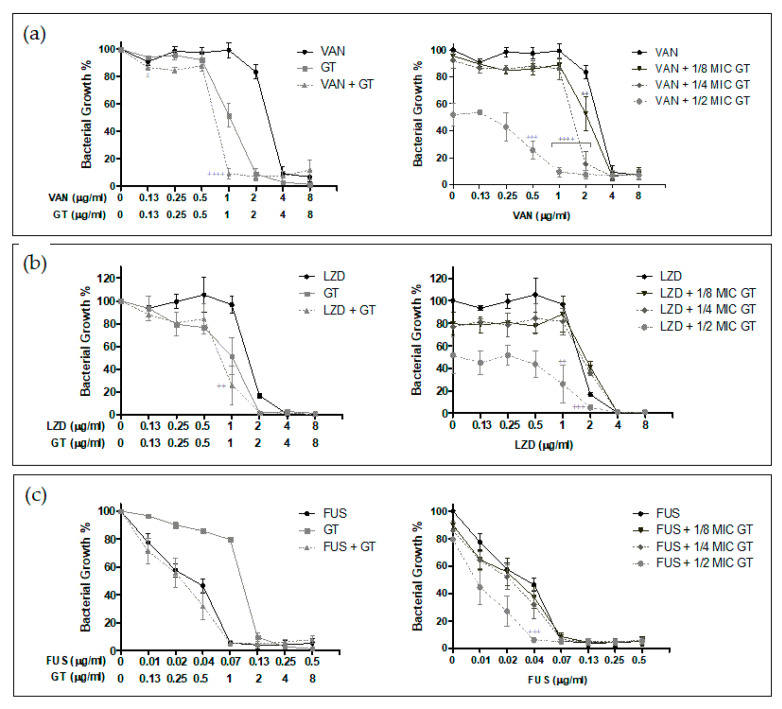
In vitro synergy assay results for MRSA/VISA ATCC 700699 strain. The effect of GT and different antibiotics as monotherapy or in combination was tested against *S. aureus* as described in Materials and Methods. (**a**) Combination of GT (GT) and vancomycin (VAN). (**b**) Combination of GT + linezolid (LZD). (**c**) Combination of GT + fusidic acid (FUS). For each panel, the concentration of each compound and the mean ± s.d. of the percentage of bacterial growth obtained in the experiments are indicated. The symbols correspond to the different levels of synergy as indicated in Materials and Methods, calculated using the CalcuSyn software as indicated in Materials and Methods: (++) Moderate synergism, (+++) Synergism, and (++++) Strong synergism. MIC of GT = 2 µg/mLfor *S. aureus* ATCC 700699. *n* = 3.

As shown in Figure 4 and Figure 5, several of the tested combinations of GT with antibiotics were found to be synergistic, and the antimicrobial activity of single compounds was increased.

For the MSSA strain, the results in Figure 4 show synergistic GT activity with all the antibiotics tested except for cloxacillin. In the case of the combination with vancomycin, a drastic decrease in the growth percentage for all combinations was observed, resulting in strong and very strong synergism. In the tested combinations of GT with linezolid, the reduction of the growth percentage was observed to a lesser extent, but again, all the concentrations tested showed synergistic effects—from nearly additive to very strong synergism, the best combination being½ MIC of GT (2 µg/mL) plus concentrations from 0.13 to 2 µg/mL of linezolid. GT in combination with fusidic acid showed synergy when using 0.07 µg/mLof fusidic acid with 1 and 2 µg/mL of GT. It also showed synergy when combining 2 µg/mL of GT with 0.01 µg/mL of fusidic acid, where a large reduction in the growth percentage was seen. Notably, such a low concentration of fusidic acid only showed around 70% growth inhibition. Finally, 2 µg/mL GT showed strong synergism in combination with 0.02 and 0.04 µg/mL of fusidic acid.

Regarding the MRSA strain, synergistic effects of GT in combination with vancomycin, linezolid, and fusidic acid were demonstrated (Figure 5). In this case, fusidic acid at 0.04 µg/mL showed synergy when combined with GT at half the MIC for this strain (1 µg/mL). GT at 1 µg/mL presented moderate synergism when combined with 1 µg/mL of linezolid and synergism when combined with 2 µg/mL (linezolid MIC for this strain). For the MRSA strain, the combination of compounds with the greatest synergistic effects was GT plus vancomycin. Synergy was observed when combining 0.5 µg/mL of vancomycin with 1 µg/mL of GT, although the growth percentage exceeded 20%. At higher concentrations of vancomycin, a very strong synergism and greater reduction in the growth percentage were found when combining 1 µg/mL of GT with 1 µg/mL of vancomycin and when combining 0.5–1 µg/mL of GT with 2 µg/mL of vancomycin.

### 2.3. Mutant Prevention Concentration of GT and Vancomycin Alone or in Combination against S. aureus

Next, we determined the mutant prevention concentration (MPC) of GT and vancomycin alone or in combination. These experiments were only carried out with *S. aureus* ATCC 700699, GT, and vancomycin since GT has been shown to be an anti-staphylococcal compound against this methicillin-resistant and vancomycin-intermediate strain and shows synergy in combination with vancomycin. It should also be noted that the study of MRSA and VISA strains is more relevant for clinical and pharmaceutical settings.

All MPC determinations were made in triplicate and the results were identical. Table 1 reflects the MPCs of antimicrobials alone and in combination with vancomycin against the MRSA strain.

The MPC of vancomycin was halved when it was combined with a very low concentration of GT, specifically the MIC of GT for this strain (2 µg/mL).

### 2.4. In Vivo Antibiotic Efficacy of GT as Monotherapy and in Combination with Vancomycin against S. aureus in C. elegans Model

The antibiotic efficacy of GT as monotherapy and in combination with vancomycin against MRSA was evaluated using a *C. elegans* infection model. The treatments selected for this survival study were those that resulted in synergistic activity in the in vitro experiments for MRSA.

The synchronization method was optimized to obtain a large number of nematodes at the same developmental stage for the survival assays. For this purpose, culture plates with a high content of gravid worms and eggs were used. The best performance was obtained when 2 plates were synchronized, distributing the M9 buffer with the worms in several 15 mL falcon tubes so that they contained a pellet of about 3 mm after centrifugation. The addition of a bleaching solution for this pellet is sufficient if the exposure time is controlled.

**Figure 6:**
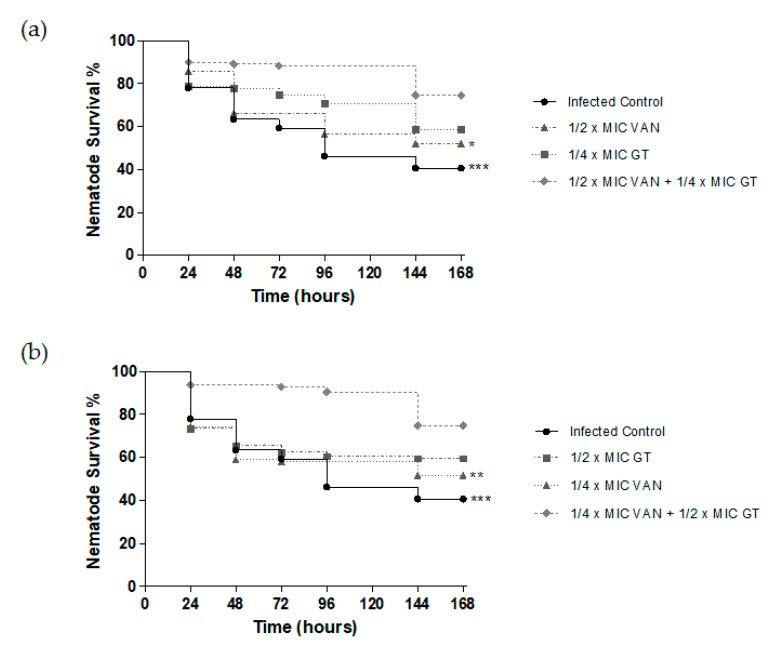
Survival curves of in vivo assay with *Caenorhabditis elegans* MRSA infection model that was treated with GT, vancomycin, and combinations of both. The survival percentage of the nematodes is represented for each treatment and for the infected control every 24 h for 7 days. (**a**) Results of monotherapy treatment with ½ MIC of vancomycin, ¼ MIC of GT, and the combination of these two concentrations. (**b**) Results of monotherapy treatment with ¼ MIC of vancomycin, ½ MIC of GT, and the combination of these two concentrations. *, **, *** Statistically significant differences (*p* < 0.05, 0.01, or 0.001, respectively) regarding the antibiotic combination performed as indicated in Materials and Methods. *n* = 4.

A significant survival rate reduction was observed in *C. elegans* infected with MRSA using liquid-based assays (Figure 6), obtaining around 40% survival. This reduction was diminished when GT and vancomycin treatments were used both in monotherapy and in combination.

Survival curves were obtained using the GraphPad Prism program (Figure 6). It was observed that the synergistic effect seen in vivo for two of the combinations produced a higher percentage of survival of the nematodes compared to the effect of the antimicrobials in monotherapy. The combination of ½ MIC of vancomycin (2 µg/mL) with ¼ MIC of GT (0.5 µg/mL) resulted in 74.55% survival of nematodes, being 58.58% for GT and 52.11% for vancomycin at the same concentrations in monotherapy. On the other hand, the combination of ¼ of vancomycin MIC (1 µg/mL) with ½ of GT MIC (1 µg/mL) resulted in 74.80% survival of nematodes. The same concentrations in monotherapy resulted in 59.60% survival for GT and 51.79% for vancomycin. Statistical analysis of the survival assay revealed that the results were statistically significant when comparing the GT plus vancomycin combination with the infected control and with the effect observed at the same concentration of vancomycin in monotherapy.

**Figure 7:**
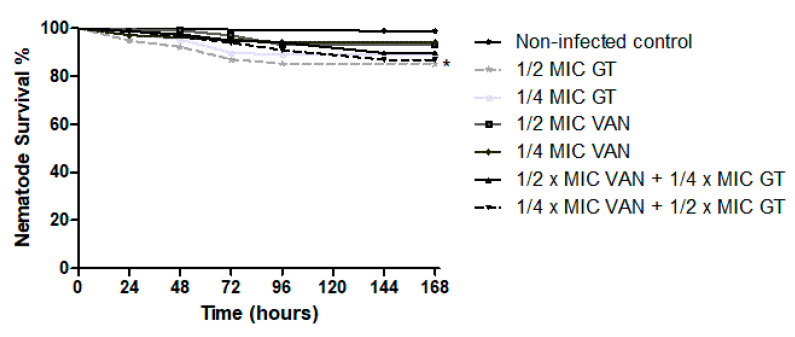
Toxicity test in non-infected *C. elegans* treated with GT, vancomycin, and combinations of both. The survival percentage of the nematodes is represented for each treatment and for the non-infected control every 24 h for 7 days. Results of monotherapy treatment with ½ MIC of vancomycin, ½ MIC of GT, ¼ MIC of vancomycin, ¼ MIC of GT, and the combination of two concentrations. *, Statistically significant differences (*p* < 0.05) regarding the non-infected control. *n* = 4.

The toxic effect of GT and vancomycin alone or in combination was also studied in *C. elegans* at the concentrations that had been tested in the infection experiments (Figure 7). The survival percentage for vancomycin was 93.00% and 94.74% for 2 and 1 µg/mL, respectively. Regarding GT alone, survival was slightly reduced, 85.22% and 88.98% for 1 and 0.5 µg/mL, respectively, although it was not significantly different to vancomycin. Moreover, with respect to combinations, 89.74% survival was shown for 2 µg/mLof vancomycin plus 0.5 µg/mL of GT. Additionally, 86.87% of nematodes survived in the case of the combination of 1 µg/mL of vancomycin with 1 µg/mL of GT. Thus, these results indicate that GT might show a slight toxic effect at the tested concentrations, although this effect is not significantly different from that observed in vancomycin.

## 3. Discussion

*S. aureus* is one of the leading causes of skin and soft tissue infections in all age groups. There is also evidence of an increase in community-acquired methicillin-resistant *S. aureus* infections (CA-MRSA). CA-MRSA infections can be difficult to treat due to resistance to antibiotics, and their infection rate is on the rise worldwide [27]. Therefore, the study of new antibiotics as monotherapy or in combination with existing treatments is necessary to be able to deal with these infections.

Since its discovery, GT has sparked interest in exploiting its antimicrobial activities, which has been tested against viral, fungal, and bacterial pathogens [24,28,29,30]. Our findings reveal the antimicrobial activity of GT against *S. aureus* strains both sensitive and resistant to methicillin and with reduced susceptibility to vancomycin (that is, MRSA/VISA strains). Regarding the MSSA strain, the only trial performed with the same strain that has been found in the literature [25] resulted in a MIC = 3.98 μg/mL, a value practically equivalent to ours (MIC = 4). Some researchers have studied the effect of GT against different MRSA strains, obtaining a MIC range between 0.5–4 μg/mL [25,31,32], confirming the validity of the result found in this study for the MRSA ATCC 700699 strain (MIC = 2 μg/mL). Importantly, our results provide novel observations and extend previous analyses by showing that GT presents activity not only against MRSA but against a MRSA/VISA strain.

The frequent use of vancomycin as the main agent to treat MRSA infections is a direct consequence of the worldwide spread of multidrug resistant MRSA clones during the last decades. This increased selective pressure has resulted in the emergence of MRSA isolates with reduced susceptibility to vancomycin and, more recently, in the emergence of VISA strains with a high level of antibiotic resistance [33]. For the vast majority of cases, VISA strains have emerged in patients with MRSA infections undergoing prolonged vancomycin therapy, which often ended in treatment failure [34,35,36,37]. At present a large number of clinical strains of *S. aureus* with reduced susceptibility to vancomycin have been reported, including heteroresistant VISA (resistant subpopulations among the total bacterial population of the strain, which can be selected by treatment) and VISA [38].

For all these reasons, the strain we selected for the assays in this study was ATCC 700699, a MRSA and VISA strain. The results obtained are novel since there is no scientific evidence to determine the effect of GT for VISA strains. Although the molecular determinants of resistance are not completely determined, it is clear that the sequential acquisition of point mutations can lead to resistance [36]. Here we demonstrate that the combination of GT with vancomycin decreases its MPC (MIC of the least-susceptible, single-stepmutant), thus reducing the lowest drug concentration required to block the growth of the least susceptible cell present in high density bacterial populations.

For patients with recurrent MRSA infections, minimization of vancomycin exposures (possibly through use of aggressive debridement or administration of non-glycopeptide antimicrobials, when appropriate) should be considered to prevent the emergence of VISA infection [35]. Therefore, it could be argued that combination therapy using a lower concentration of vancomycin may be appropriate in an attempt to avoid the selection of resistant mutants in severe *S. aureus* infections. Vancomycin administered in suitable doses together with other active agents may still present a relatively safe option for the treatment of patients with MRSA infections once the clinical isolate has been appropriately tested [36].

We studied the synergistic effect of the combination of GT with different antibiotics employed in the clinics for the treatment of *S. aureus* infections. The in vitro results show that GT is capable of enhancing the effect of these drugs, making it possible to reduce the concentration at which they are effective for MSSA and MRSA/VISA strains. The results are especially encouraging when vancomycin is combined with GT, as a powerful synergistic effect is obtained by reducing the vancomycin concentration to ½ or even ¼ of the MIC, still obtaining a powerful antibiotic effect. The in vivo results in the *C. elegans* infection model validate the in vitro findings and demonstrate the potentiating effect of GT when combined with vancomycin, resulting in a significantly superior antibiotic effect when certain concentrations of GT and vancomycin are combined. The mechanistic explanations for this synergistic effect are currently unknown and will require further experimental testing. Several targets of *S. aureus* have been found to be affected by vancomycin altering cell wall synthesis, permeability, and RNA synthesis, and different mutations in genes involved in these processes have been proposed to mediate resistance [33]. GT has been found to directly bind to Cys residues of proteins, modifying their function in both eukaryotic and prokaryotic cells. In addition, GT-induced reactive oxygen species can affect proteins, lipids, and DNA. Thus, it might be possible that the effect of GT on these molecules might modulate vancomycin resistance in *S. aureus* and thus could promote a synergistic effect between both antimicrobials. However, this is a hypothesis that will require experimental validation.

GT at the concentrations studied is barely toxic to *C. elegans*. Nevertheless, since GT has been shown to be toxic to different mammalian cell types [39], a detailed toxicology study involving skin cells (the main tissue where serious infections occur by resistant *S. aureus* [27]) as well as mammal in vivo models will be required to support the feasibility of the clinical application of the lowest effective doses of GT combined with vancomycin. In addition, it might be important to consider the development of pharmaceutical forms that do not cross the corneal layer or that have little penetration capacity and, on the other hand, the use of site-specific drug-delivery vehicles to reduce GT entry into systemic circulation.

Pending thesolving of these potential limitations for future clinical development, our results show that GT presents antimicrobial activity in vitro and in vivo against a clinically relevant MRSA/VISA *S. aureus* strain. Importantly, its ability to synergistically enhance the effect of vancomycin presents promising prospects for new drug development to help in treating drug resistant strains and to reduce the emergence of vancomycin-resistant strains.

## 4. Materials and Methods

### 4.1. Staphylococcus aureus Strains

MSSAATCC 29213 and MRSA/VISA ATCC 700699 bacteria were provided by Dr. Antonio Rezusta from Hospital Universitario Miguel Servet (Zaragoza, Spain). They were used to study the effect of GT (Enzo Life Sciences, Inc., Farmingdale, NY, USA) through antimicrobial susceptibility, synergism, and in vivo infection model assays.

The bacterial inoculum was freshly prepared for each independent experiment. *S. aureus* was grown in Luria–Bertani (LB, Sigma-Aldrich, St. Louis, MO, USA) overnight at 37 ± 2 °C with shaking at 170 rpm. It was centrifuged and washed with PBS and resuspended again in PBS.

The bacterial load required for each experiment was adjusted by determining the optical density at 600 nm (OD600) with a spectrophotometer (OD600 DiluPhotometer, IMPLEN, Munich, Germany), based on our own line of CFU/mL-OD600 calculated for these strains.

### 4.2. Antimicrobial Susceptibility Assays

The clinical microbiology procedure recommended by EUCAST [40] and the Clinical and Laboratory Standards Institute (CLSI) [41] was followed to perform the agar diffusion method. A representative antibiotic with known antimicrobial efficacy was chosen for each particular strain as antimicrobial control. Commercial disks (Thermo Scientific™ Oxoid™, Thermo Fisher Scientific, Waltham, MA, USA) were used. A control with cefoxitin (30 μg) was used for the methicillin-sensitive strain and the same control in addition to fusidic acid (10 μg) for the methicillin-resistant strain. In the case of GT, non-impregnated disks (Suministros Clínicos Lanau, Zaragoza, Spain) were used. These were deposited on the plate and 10 µg GT (Enzo Life Sciences, Inc.; 10 µL of 1000 µg/mL solution) was added to them. The non-impregnated disks were also used to add 10 μLof the GT vehicle (water: methanol 1.2:1) as a control to rule out the effect of methanol on microorganisms. The experiments were carried out in triplicate for each strain.

The broth microdilution assay to determine the MIC was performed in triplicate in accordance with CLSI [42] for *S. aureus* ATCC 29213 and ATCC 700699. It was performed using two-fold serial dilutions of GT from 125 to 0.49 µg/mL in LB or Mueller-Hinton (MH), adding the bacterial suspension at a final concentration of 5 × 10^5^ CFU/mL. Since similar results were found using LB and MH, LB was employed for the rest of the experiments.

The microdilution plates were incubated at 37 ± 2 °C for 24 h. Then, the OD630 of each well was measured with a spectrometer plate reader (BioTek Synergy HT) to determine the concentration exhibiting in vitro inhibition of *S. aureus* growth. The MIC was determined in triplicate using cloxacillin (0.04–2 µg/mL), linezolid (0.13–8 µg/mL), fusidic acid (0.01–0.5 µg/mL), and vancomycin (0.07–8 µg/mL) as antibiotic controls (Sigma-Aldrich).

The results were statistically analyzed using GraphPad Prism version 5.0 for Windows, GraphPad Software, San Diego, CA, USA (www.graphpad.com). The absorbance data obtained in each well were compared with the controls. At each absorbance value, the OD630 value obtained in the negative control, which only contained the medium, was subtracted, and the percentage of growth or viability was calculated with respect to the growth in the absence of antimicrobial. One-way ANOVA of the data corresponding to GT was also performed with respect to the positive control.

### 4.3. Antimicrobial Synergy Study—Checkerboard Testing

The synergistic effect of GT with anti-staphylococcal drugs (cloxacillin, vancomycin, fusidic acid, and linezolid) against *S. aureus* was tested by a checkerboard assay. This test wasused to determine the impact on potency of the combination of antibiotics in comparison to their individual activities.

Checkerboard synergy testing was performed by the microbroth dilution method in triplicate, as previously described [43]. For this purpose, a two-dimensional array of serial concentrations of test compounds wasused. Both GT and antibiotics were used to test synergy in different combinations and concentrations: it was started with twice or four times the MIC, and serial dilutions were prepared in the corresponding wells, creating a gradient of concentrations for each compound:GT (0.13–8 µg/mL) was combined with vancomycin (0.13–8 µg/mL), linezolid (0.13–8 µg/mL), and fusidic acid (0.01–0.5 µg/mL) for *S. aureus* ATCC 700699 strain, while GT was combined with vancomycin (0.07–4 µg/mL), linezolid (0.13–8 µg/mL), fusidic acid (0.01–0.5 µg/mL), and cloxacillin (0.02–1 µg/mL) for *S. aureus* ATCC 29213 strain.

The bacterial suspension was added at a final concentration of 5 × 10^5^ CFU/mL. Growth control and sterility control were included in each test panel. Microtiter plates were incubated at 37 °C overnight before the measurement of the absorbance value. The OD630 was measured using a 96-well plate reader (BioTek Synergy HT). We used the percent growth calculated with the OD630 data as drug effect to process the data using the CalcuSyn software (CalcuSyn software version 2.1 for Windows, Biosoft: Chou, 1996–2007). The CalcuSyn program is based on the Chou–Talalay method for drug combination [44], which calculates the combination index (CI). The CI is a quantitative representation of pharmacological interactivity that takes into account both the potency and the shape of the dose response curve. The CI was generated by the CalcuSyn software over a range of fraction of cells affected levels at different growth inhibition percentages and was interpreted in accordance with Table 2.

The more broadly defined criteria suggest that the combination index, CI < 1, = 1, and > 1 indicate synergism, additive effect, and antagonism, respectively.

### 4.4. Determination of the Mutant Prevention Concentration

The MPC was described by Dong et al. [46] as a novel in vitro measurement of antimicrobial susceptibility and takes into account the probability of mutant subpopulations being present in high density bacterial populations.

A slightly modified previously described method was used [47]. We performed a microdilution method in 96-well plates similar to the checkerboard assay to create a gradient of concentrations in LB for GT and vancomycin alone or in combination.

According to the MIC of antimicrobial agents, 50 μL of *S. aureus* ATCC 700699 culture containing about 2.0 × 10^9^ CFU wasplated on series wells containing two-fold dilution of final concentrations from 256× MIC of vancomycin and 128× MIC of GT. A positive growth control without antimicrobial and a negative control with only LB were used.

The plates were incubated at 37 °C for 24 h. Then, MPC was recorded as the lowest antimicrobial concentration for each treatment alone and in combination that prevented bacterial growth. The bacterial growth was assessed by seeding each well in LB agar plates. The determination of the MPC for each antimicrobial and combination was the lowest concentration that did not show bacterial growth in any of the three experiments.

### 4.5. C. elegans Culturing and Synchronization

*C. elegans* SS104 was used to develop an in vivo infection model assay. The SS104 strain is very useful for producing large populations of worms that lack a germ line, due to the glp-4 mutation that makes worms unable of generating progeny when they are cultured at 25 °C [48]. This mutant can be obtained from the Caenorhabditis Genetics Centre (http://www.cbs.umn.edu/research/resources/cgc).

*C. elegans* worms were propagated on nematode growth medium (NGM) agar plates (NGM Lite, US Biological Life Sciences, Swampscott, MA, USA) supplemented with streptomycin 100 µg/mL (Sigma-Aldrich) and kanamycin 50 µg/mL (Sigma-Aldrich) at 20 °C, using *E. coli* OP50 (Caenorhabditis Genetics Centre) as source of food.

Worms are in different developmental stages in culture; therefore, they must be synchronized for further use. The synchronization process consists of obtaining only viable eggs. Cultured worms in NGM agar plates were washed with enough M9 buffer (Na_2_HPO_4_, 6 g; NaCl, 5 g; KH_2_PO_4_, 3 g; distilled H_2_O, 1 L; and 1 mL of MgSO_4_ 1M) to pick up all worms and unstick eggs from agar plates. Worm suspension in M9 buffer was transferred to 15 mL Falcon tubes. The suspension was centrifuged (Beckman Coulter Allegra X-15) at 650× *g* for 2 min. The supernatant was discarded and 2 mL of M9 buffer with pool of different stage worms and eggs wasleft. By synchronization method, the larvae and adult worms were killed and the cuticle of *C. elegans* was weakened through a bleaching solution to release the eggs of the gravid worms. Bleaching solution containing 600 µL of 1M NaOH and 600 µL of commercial NaClO was freshly prepared and added to the worms. Worms with bleaching solution were vortexed for ten seconds and an aliquot was taken to check the worms under a dissecting microscope (Leica DMi1 u Olympus IX81). If worms remained alive or the cuticle of most worms had not been broken, more 10 s vortex events up to a maximum contact time of 10 min could be required. Higher concentration of NaOH might be needed to get the breakage of the cuticle if the suspension contained too many worms. Watching worms regularly on a microscope provided the best information to know when the bleaching process must be stopped. At this point, the 15 mL Falcon tube was filled with M9 buffer and centrifuged 2 min at 650× *g* and repeated twice more in order to reduce the amount of NaOH and NaClO remaining. The supernatant of washing M9 was discarded until only 0.5 mL with eggs pellet stayed in the tube.

Egg pellet was resuspended and plated on a NGM agar plate without *E. coli* OP50 to allow eggs to hatch and reduce developmental differences in new larvae due to different egg age. From this point on, the plates were incubated at 25 °C so that the worms did not develop a germ line when they grew and were not capable of procreating. *E. coli* OP50 was added to the NGM agar plate 24 h later. As a result of synchronization, a pool of similar aged and developed worms was obtained.

### 4.6. C. elegans L4 Larvae Culture and Infection

L1 larvae obtained from synchronization were cultured at 25°C until worms developed to L4 stage. *C. elegans* L4 larvae were used to perform a liquid-based assay to infect them with *S. aureus,* as previously described [49].

For the liquid-based assay, a 94-well flat-bottom plate was filled with *S. aureus* in a liquid medium (80% M9 buffer, 20% *S. aureus* ATCC700699 culture) and approximately 15–20 synchronized young adult nematodes were transferred into each well.

The *S. aureus* overnight culture in LB approximately contained 1 × 10^9^ CFU/mL. It was centrifuged at 3500 rpm for 10 min to discard the supernatant and resuspend in 4 mL of PBS. This process was repeated twice.

### 4.7. C. elegans Survival Assay

Different concentrations of GT, vancomycin, and combinations were added to the 96-well plates containing infected or non-infected *C. elegans* to evaluate the effectivity and toxicity of the treatments. Wells containing only *C. elegans*, *E. coli* OP50, and M9 buffer served as controls. The plates were incubated at 25 °C and worms’ survival was monitored every 24 h for 7 days following exposure to the pathogen and treatment. Four independent experiments were performed. In each experiment, the total number of worms counted for each group was ≥30.

Survival rate was calculated by counting the number of living nematodes remaining in each group at the corresponding time with a microscope (Leica DMi1 u Olympus IX81). Distinguishing living nematodes from dead ones is a simple process. Live worms constantly move around and take on rounded shapes. When the worms began to feed on *S. aureus* and it began to colonize their intestinal tract, thickening and wrinkling of the cuticle could be observed. Dead nematodes are easy to distinguish, as they are dark and stiff, often taking a right-angle shape.

Statistical analysis for *C. elegans* survival assay was performed using Kaplan–Meier curves and log-rank and Gehan–Wilcoxon tests employing GraphPad Prism software. In this study there was aneed for multiple comparisons due to the fact that we were comparing several survival curves at once. In this case, we wanted to drill down and compare curves twice at time. To obtain this statistical analysis we performed one-way ANOVA followed by Bonferroni’s post-hoc comparisons tests.

## Figures and Tables

**Table 1:** Mutant Prevention Concentration obtained for GT, vancomycin, and combination of both against *S. aureus* ATCC 700699. *n* = 3.

Compound	Individually		Combination
Vancomycin	64	0	32
Gliotoxin	0	256	2

Mutant Prevention Concentration (µg/mL).

**Table 2:** Combinatory index values, recommended symbols, and descriptions for classifying synergism or antagonism using the Chou–Talalay method.

Range of combination index (CI)	Symbol	Description
<0.1	+++++	Very strong synergism
0.1–0.3	++++	Strong synergism
0.3–0.7	+++	Synergism
0.7–0.85	++	Moderate synergism
0.85–0.90	+	Slight synergism
0.90–1.10	±	Nearly additive
1.10–1.20	–	Slight antagonism
1.20–1.45	– –	Moderate antagonism
1.45–3.3	– – –	Antagonism
3.3–10	– – – –	Strong antagonism
>10	– – – – –	Very strong antagonism

Source: CalcuSyn manual, Biosoft, 2006 [45].

## Data Availability

Data is contained within the article or supplementary material.
